# Interleukin-1β mediates high glucose induced phenotypic transition in human aortic endothelial cells

**DOI:** 10.1186/s12933-016-0358-9

**Published:** 2016-03-05

**Authors:** Dong-Dong Zhu, Ri-Ning Tang, Lin-Li Lv, Yi Wen, Hong Liu, Xiao-Liang Zhang, Kun-Ling Ma, Bi-Cheng Liu

**Affiliations:** Institute of Nephrology, Zhongda Hospital, Southeast University School of Medicine, Nanjing, 210009 China

**Keywords:** High glucose, Endothelial damage, Interleukin-1β, PKC

## Abstract

**Background:**

Previous studies have shown that high glucose (HG) induced endothelial cell (EC) damage via a phenotypic transition of EC. There is increasing evidence suggesting the role of inflammatory cytokines in mediated HG-induced EC damage. However, little is known about the potential role of interleukin-1β (IL-1β) in the process. The aim of present study was to investigate whether IL-1β mediated HG–induced phenotypic transition in human aortic endothelial cells (HAECs) and to determine the possible underlying mechanism.

**Methods:**

Primary HAECs were exposed to normal glucose (NG, 5.5 nM), high glucose (HG,30 nM), IL-1β (10 ng/ml), HG + IL-1β (10 ng/ml) and HG + anti-IL-1β antibodies (1000 ng/ml) or HG + IL-1β small interfering RNA (siRNA). Pathological changes were investigated using confocal microscopy and electron microscopy. Confocal microscopy was performed to detect the co-expression of CD31 and fibroblast specific protein 1 (FSP1). To study the effect of protein kinase C-β (PKCβ) activation on IL-1β in HAECs, HAECs were stimulated with 30 nM PMA (PKCβ activator) and 0.3 μM PKCβ inhibition (LY317615) for 48 h in the NG or HG group. The expressions of PKCβ and IL-1β were detected by RT-PCR and Western blot. And the concentration of IL-1β in the supernatant of HAECs was measured by ELISA. The expressions of FSP1, a-SMA and CD31 were detected by Western blot.

**Results:**

It was shown that the HG resulted in significant increase in the expressions of PKCβ and IL-1β in dose-and time-dependent manners. The HG or exogenous IL-1β alone inhibited the expression of CD31 and markly increased the expressions of FSP1 and α-SMA. Furthermore, we observed that the HG and IL-1β synergistically increased FSP1 and a-SMA expressions compared with the HG or IL-1β alone group (*P* < 0.05). Confocal microscopy revealed a colocalization of CD31 and FSP1 and that some cells acquired spindle-shaped morphologies and a loss of CD31 staining. Electron microscopy showed that the HG resulted in the increased microfilamentation and a roughened endoplasmic reticulum structure in the cytoplasm. However, the changes above were attenuated by the intervention of anti-IL-1β antibodies or IL-1β siRNA (*P* < 0.05). In addition, the PMA induced the expressions of PKCβ and IL-1β in HAECs. The PKCβ activation may mediate the effect of the HG on IL-1β production, which could be attenuated by the PKCβ selective inhibitor (LY317615) (*P* < 0.05).

**Conclusions:**

Our findings suggested that HG-induced phenotypic transition of HAECs might require IL-β activation via the PKCβ pathway.

## Background

Diabetes is a growing epidemic worldwide. Vascular complications of diabetes are the most serious manifestations of the disease. Endothelial cell (EC) damage is a critical and initiating factor in the development of diabetic vascular complications [1–3]. Emerging evidence has suggested that diabetes was one of the inflammatory diseases and high glucose (HG) induced inflammation is involved in the onset and progression of EC damage [4]. Furthermore, previous studies have also shown that the inflammatory cytokines promote the EC damage [5–7].

In particular, interleukin-1β (IL-1β) has been involved in the pathogenesis of diabetes [8–10]. Accordingly, IL-1β antagonist has been proposed as a promising therapeutic approach to diabetes [11, 12]. Moreover, further studies elucidated the link between IL-1β and the development of cardiovascular complications [13, 14]. Recently, Vallejo et al. [15] indicated that IL-1β correlated with EC damage in a short-term model of type 1 diabetes. Together, these findings indicated that IL-1β may play a key role to EC damage in diabetes. However, the underlying mechanism remains to be not understood.

Interestingly, multiple studies reported that IL-1β can initiate the transition of EC to mesenchymal cell [16–19]. This transition process is called endothelial to mesenchymal transition (EndMT), characterized by the loss of cell–cell adhesion, the changes in cell polarity, the reduction of EC markers, such as CD31, and the overexpression of mesenchymal cell markers, such as α-smooth muscle actin (α-SMA) and fibroblast-specific protein1 (FSP1). More recent studies demonstrated that EndMT contributed to EC damage [20–23]. Additionally, our previous studies also proved that HG could induce EC damage via EndMT [24, 25].

Therefore, the aim of this study was to investigate whether IL-1β mediates HG–induced phenotypic transition in human aortic endothelial cells (HAECs) and the underlying mechanism of IL-1β regulation.

## Methods

### Cell culture and reagents

Primary HAECs were purchased from Sciencell Research Laboratories (USA) and cultured as previously described. Briefly, cells were grown in endothelial culture medium (No. 1001, Sciencell) containing 5 % fetal bovine serum (FBS) (No. 0025), 1 % endothelial cell growth supplement (No. 1052) and 1 % penicillin/streptomycin solution (No. 0503) in 5 % CO2 at 37 °C. Passage 2–4 HAECs were expanded in monolayers in flasks or dishes. At approximately 80 % confluence, the culture medium was changed to a serum-free solution for 24 h prior to their use in all of the experiments. To examine the effect of high glucose on IL-1β secretion and phenotypic transition in HAECs, HAECs were treated with normal glucose (NG: 5.5 mM), 15 mM d-glucose, HG (30 mM d-glucose) and MN (5.5 mM NG + 24.5 mM mannitol) for 48 h. And to identify the effect of IL-1β on phenotypic transition in HAECs, the cells were cultured in the presence of IL-1β(Peprotech, 10 ng/ml),anti-IL-1β antibodies(R&D System, 1000 ng/ml)or IL-1β siRNA (Santa Cruz, sc-39615 IL-11β siRNA(h); sc-45064 siRNA Reagent System) for 48 h. To examine the effect of PKCβ activation on IL-1β production, HAECs were stimulated with 30 nM phorbol 12-myristate 13-acetate (PMA, Sigma) [10] in NG group, and were incubated with HG/NG containing the PKCβ selective inhibitor LY317615 (Selleck, 0.3 uM).

### Enzyme linked immuno-sorbent assay (ELISA)

The cell supernatant was collected, and the IL-1β production was measured using a commercially available ELISA kit (R&D System) according to the protocol described by the manufacturer.

### Real-time PCR

Total RNA was extracted using RNAiso Plus according to the manufacturer’s directions (TAKARA, China). RNA concentration and purity were confirmed with a Nanodrop 2000 (Thermo, USA). Samples with a relative absorbance ratio at 260/280 between 1.8 and 2.0 were used. All of the RNA samples were reverse transcribed (Applied Biosystems, USA). Quantification of specific mRNAs was performed using an ABI Prism 7300 Sequence Detection System (Applied Biosystems, USA) with the SYBR Green Real-time PCR Kit (TAKARA, China). The following oligonucleotide primer sequences were used: IL-1β:forward 5′,T G A A A T G A T G G C T T A T T A C A G T G G 3′, reverse 5′,G T A G T G G T G G T C G G A G A T T C G T A G 3′; PKCβ:forward 5′,G A A A T T T G A G A G G G C C A A G A 3′, reverse 5′,C C C A G C A C C A T T A G G A A G T T 3′, (designedand synthesized by Generay, China). Relative mRNA amounts were normalised to GAPDH and calculated using the standard curve method. In brief, the pre-PCR product of each gene was used as the standard. The standard curve was established with a tenfold serial dilution of the product and was included in all PCR runs. The ratio of targetgene housekeeping was used to evaluate the expression level of each gene. Control consisting of ddH2O were negative in all runs.

### Western blot analysis

Total cellular protein was extracted to evaluate the levels of CD31, FSP1, α-SMA, IL-1β and PKCβ. Equal amounts of protein obtained from each lysate were electrophoresed in a 4–20 % SDS–polyacrylamide gel and transferred onto nitrocellulose membranes (Pall, USA) by electroblotting. The blots were incubated overnight with primary antibodies against CD31 (sc-1506, Santa Cruz, CA), FSP1 (ab-27957, Abcam), a-SMA (ab-5694, Abcam), IL-1β (MAB601, R&D System) and PKCβ (GTX113252, GeneTex) followed by horseradish peroxidase-labelled secondary IgG (Santa Cruz). Signals were detected using an advanced ECL system (GE Healthcare, UK). β-actin was used as the internal control.

### Confocal microscopy

HAECs grown on coverslips were fixed in 4 % paraformaldehyde and permeabilised with 0.5 % Trition-X100. After blocking with 10 % bovine serum albumin (BSA) in phosphate-buffered saline for 1 h at room temperature, they were incubated with primary antibodies against CD31 (sc-1506), FSP1 (ab-27957) at 4 °C overnight. After incubating with secondary antibodies for 1 h at room temperature in the dark, the images were captured using a laser scanning confocal microscope (LSM 510 META, Zeiss, Germany).

### Transmission electron microscopy

Cells were fixed in 2.5 % glutaraldehyde buffer (pH 7.4). Transmission electron microscopy (TEM) was performed according to a routine fixation and embedding procedure. Thin sections were cut on a microtome, placed on copper grids, stained with uranyl acetate and lead citrate, and examined using a transmission electron microscope (JEM-1010, JEOL, Japan).

### Statistical analysis

Data were analysed by one-way analysis of variance (ANOVA**)** using SPSS 16.0 software and were expressed as the mean ± standard deviation (SD). The results of the data analysis were considered to be significant at *P* < 0.05.

## Results

### HG upregulates IL-1β expression in HAECs

**Fig. 1:**
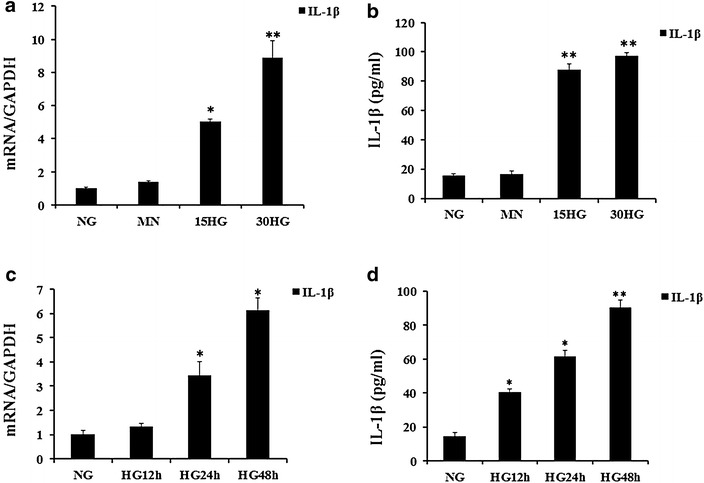
Effect of high glucose on IL-1β mRNA and protein expression in HAECs. **a**, **b** HAECs were incubated for 48 h with increasing concentrations of glucose (5.5, 15, 30 mM). Mannitol was used as a control for hyperosmolarity. **c**, **d** HAECs were grown in a 30 mM glucose medium for 12–48 h. IL-1β protein level was measured in the supernatant using ELISA and IL-1β mRNA level was assayed by quantitative RT-PCR. The data are expressed as the mean ± SD. Experiments were repeated at least three times. *NG* normal glucose (5.5 mM), *HG* high glucose (30 mM), *MN* 5.5 mM glucose + 24.5 mM mannitol. *P < 0.05 vs. MN or NG, **P < 0.01 vs. MN or NG

To demonstrate that enhanced IL-1β expression depended on the concentration and duration of HG exposure, we incubated HAECs in a media that contained 5.5, 15, or 30 mM glucose for 48 h. Mannitol was added to the control cell incubation media to equalize osmolarity. As determined by RT-PCR and ELISA, mRNA and protein expressions of IL-1β increased in response to HG exposure in a dose- and time-dependent manner (Fig. 1).

### PKCβ contributes to HG-induced IL-1β production in HAECs

**Fig. 2:**
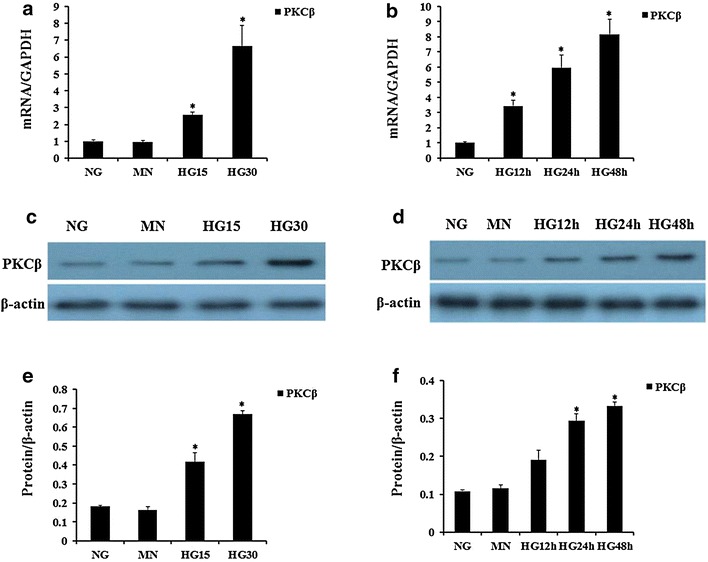
Effects of high glucose on PKCβ mRNA and protein expression in HAECs. **a, c**–**e** HAECs were incubated for 48 h with increasing concentrations of glucose (5.5, 15, 30 mM). Mannitol was used as a control for hyperosmolarity. **b**, **d**–**f** HAECs were grown in a 30 mM glucose medium for 12–48 h. PKCβ mRNA level (**a**, **b**) was assayed by quantitative RT-PCR. Representative western blots (**c, d**) and quantitative determinations of PKCβ protein levels (**e**, **f**) are presented expressed as the mean ± SD. Experiments were repeated at least three times. *NG* normal glucose (5.5 mM), *HG* high glucose 30 mM), *MN* 5.5 mM glucose + 24.5 mM mannitol. *P < 0.05 vs. MN or NG

**Fig. 3:**
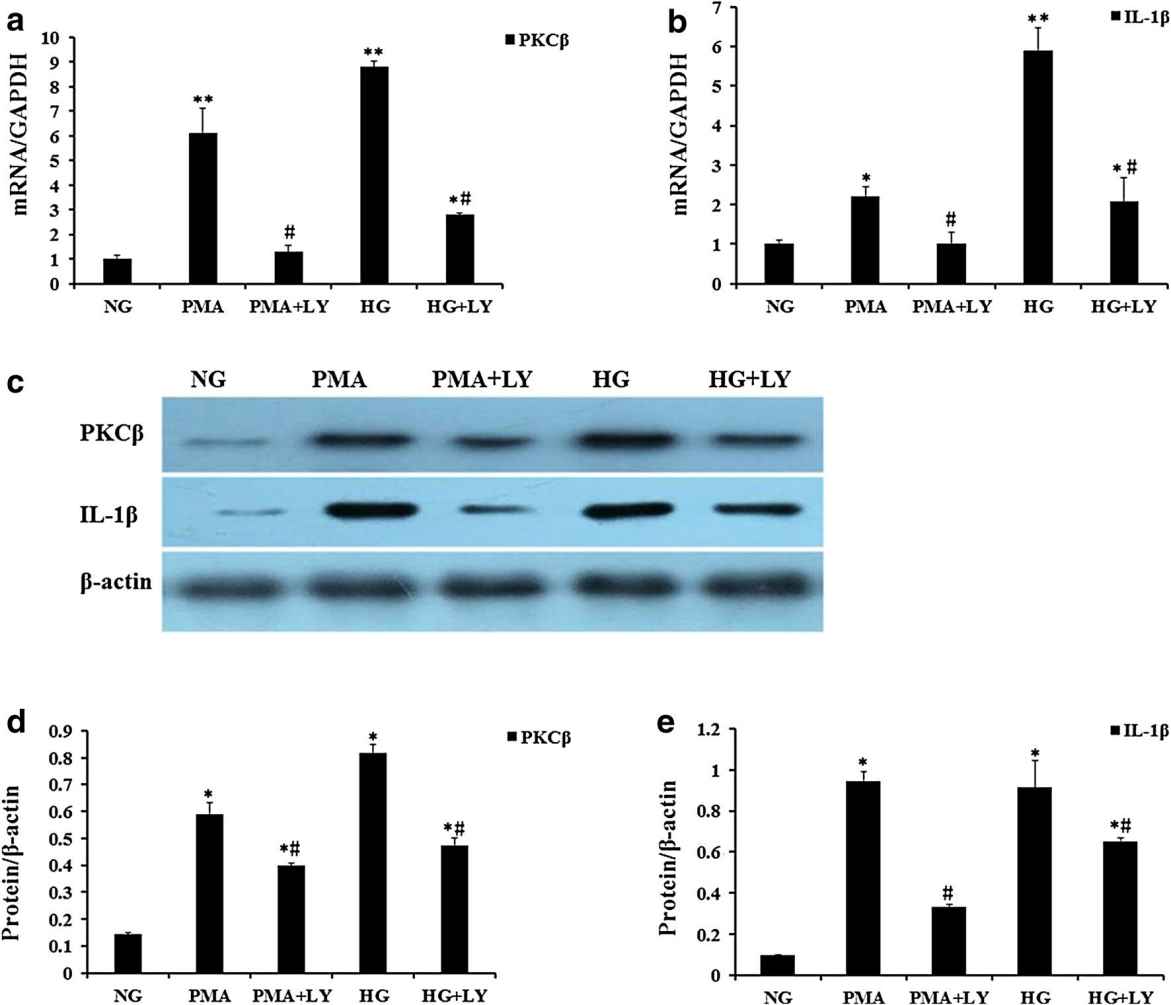
Effects of PKCβ on high glucose induced IL-1β up-regulation. Confluent cultures of HAECs were exposed to NG, HG, PMA (30 nM) and HG in the presence of the selective PKCβ inhibitors (LY317615, 0.3 μM) for 48 h. Real-time PCR analyses showed mRNA expression of PKCβ and IL-1β (**a**, **b**). Representative western blots (**c**) and quantitative determinations of PKCβ and IL-1β (**d**, **e**) are presented. The data are expressed as the mean ± SD. Experiments were repeated at least three times. *NG* normal glucose (5.5 mM), *HG* high glucose (30 mM), PMA (30 nM): phorbol 12-myristate13-acetate; LY (0.3 uM): LY317615; *P < 0.05 vs.NG, **P < 0.01 vs. NG, ^#^ *P* < 0.05 vs. HG or PMA

As shown in Fig. 2, HG has a similar effect on PKCβ expression in addition to IL-1β production. Next, to gain further insight into the mechanisms of IL-1β production in HAECs exposure to HG, we examined PKCβ and IL-1β expressions in the cells treated with PMA (PKCβ activator, 30 nM) or LY317615 (PKCβ selective inhibitor, 0.3 μM). We observed that stimulation of HAECs with PMA led to a significant up-regulation of IL-1β protein, accompanying by elevated PKCβ level, and treatment with LY317615 could inhibit the effect of HG on IL-1β (Fig. 3).

### HG and IL-1β alone or in combination resulted in the phenotypic transition in HAECs

**Fig. 4:**
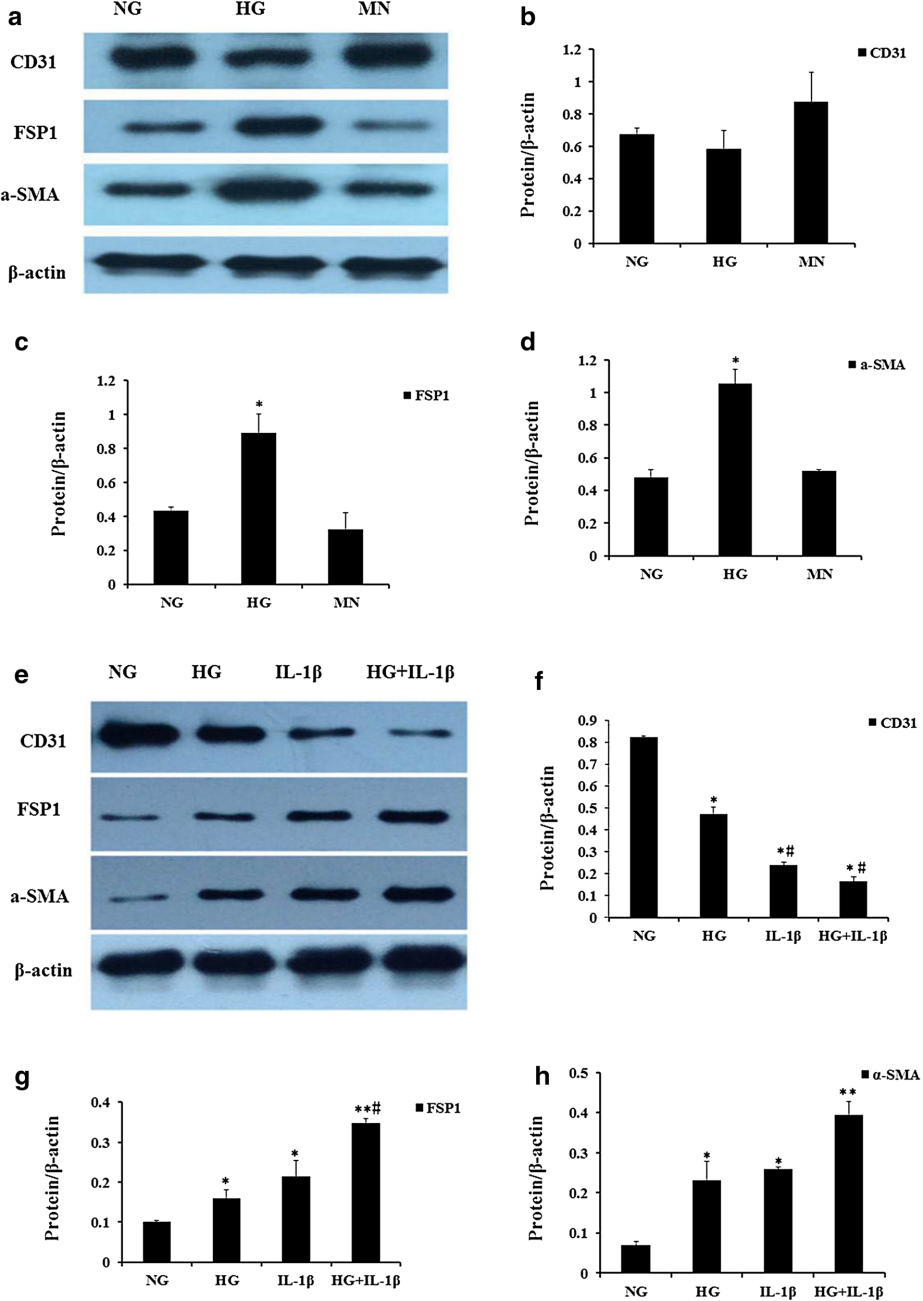
Effect of high glucose and IL-1β alone or in combination on the protein expressions of CD31, FSP1 and α-SMA in HAECs. **a**–**d** HAECs were incubated for 48 h with NG and HG. Mannitol was used as a control for hyperosmolarity. Representative western blots (**a**) and quantitative determinations of CD31, FSP1 and α-SMA protein levels (**b**–**d**) are presented. **e**–**h** HAECs were treated for 48 h with NG, HG, IL-1β (10 ng/ml)and HG in the presence of the IL-1β (10 ng/ml). Representative western blots (E) and quantitative determinations of CD31, FSP1 and α-SMA protein levels (**f**–**h**) are presented. The data are expressed as the mean ± SD. Experiments were repeated at least three times.* NG* normal glucose (5.5 mM),* HG* high glucose (30 mM),* MN* 5.5 mM glucose + 24.5 mM mannitol, IL-1β (10 ng/ml), HG + IL-1β: high glucose (30 mM) + IL-1β (10 ng/ml) *P < 0.05 vs. MN or NG, **P < 0.01 vs. NG, ^#^ *P* < 0.05 vs. HG

**Fig. 5:**
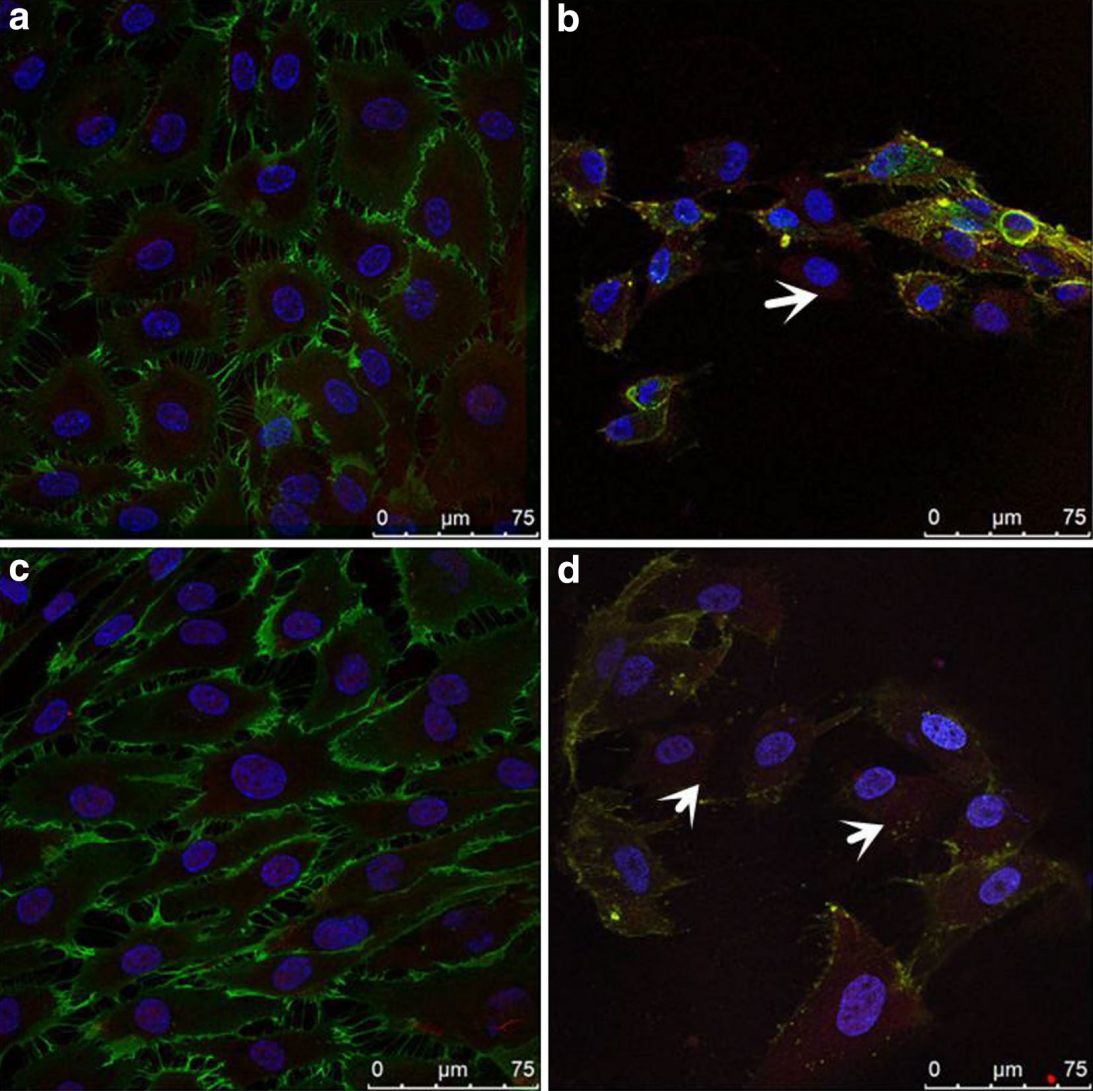
The influence of high glucose or IL-1β on immunofluorescence of CD31 and FSP1 in HAECs. Representative immunofluorescence images showing CD31 (*green*), FSP1 (*red*) labeling and DAPI (*blue*) stains nuclei. **a** Normal ECs monolayers displayed a cobble stone morphology. **b** A merge of the three images revealed some cells populations that acquired a spindle-shaped morphology and lost CD31 expression (*white arrow*). **c** HAECs exposure to IL-1β alone for 48 h acquired a spindle-shaped morphology. **d** High glucose and IL-1β in combination resulted in decreased CD31 (*the left white arrow*) and increased FSP1staining (*the right arrow*). **a** normal glucose (5.5 mM) group, **b** high glucose (30 mM) group for 48 h; **c** treatment with a normal glucose (5.5 mM) + IL-1β (10 ng/ml) treatment for 48 h, **d** treatment with a high glucose (30 mM) + IL-1β (10 ng/ml) treatment for 48 h. *Scale bar*, 75 μm

We next assessed if exogenous IL-1β or HG could induce the phenotypic transition in HAECs in a distinctive or synergistic manner. As our previous experiments demonstrated, the protein expression of FSP1 and α-SMA were progressively up-regulated, whereas the expression of CD31, an EC marker, was down-regulated in the HAECs, which were incubated with HG (30 mM) for 48 h (Fig. 4a–d). Simultaneously, a similar effect was shown in the cells treated with exogenous IL-1β (10 ng/ml). Remarkably, the protein expressions of FSP1 and α-SMA strongly increased, while CD31 expression reduced (Fig. 4e–h). We also observed the localization of CD31 and FSP1 in HAECs under confocal microscopy. As shown in Fig. 5a–d, HAECs treated with 10 ng/mL IL-1β or HG (30 mM) for 48 h acquired FSP1 staining and lost CD31 staining compared to the control cells.

### Blocking IL-1β inhibited HG-induced phenotypic transition in HAECs

**Fig. 6:**
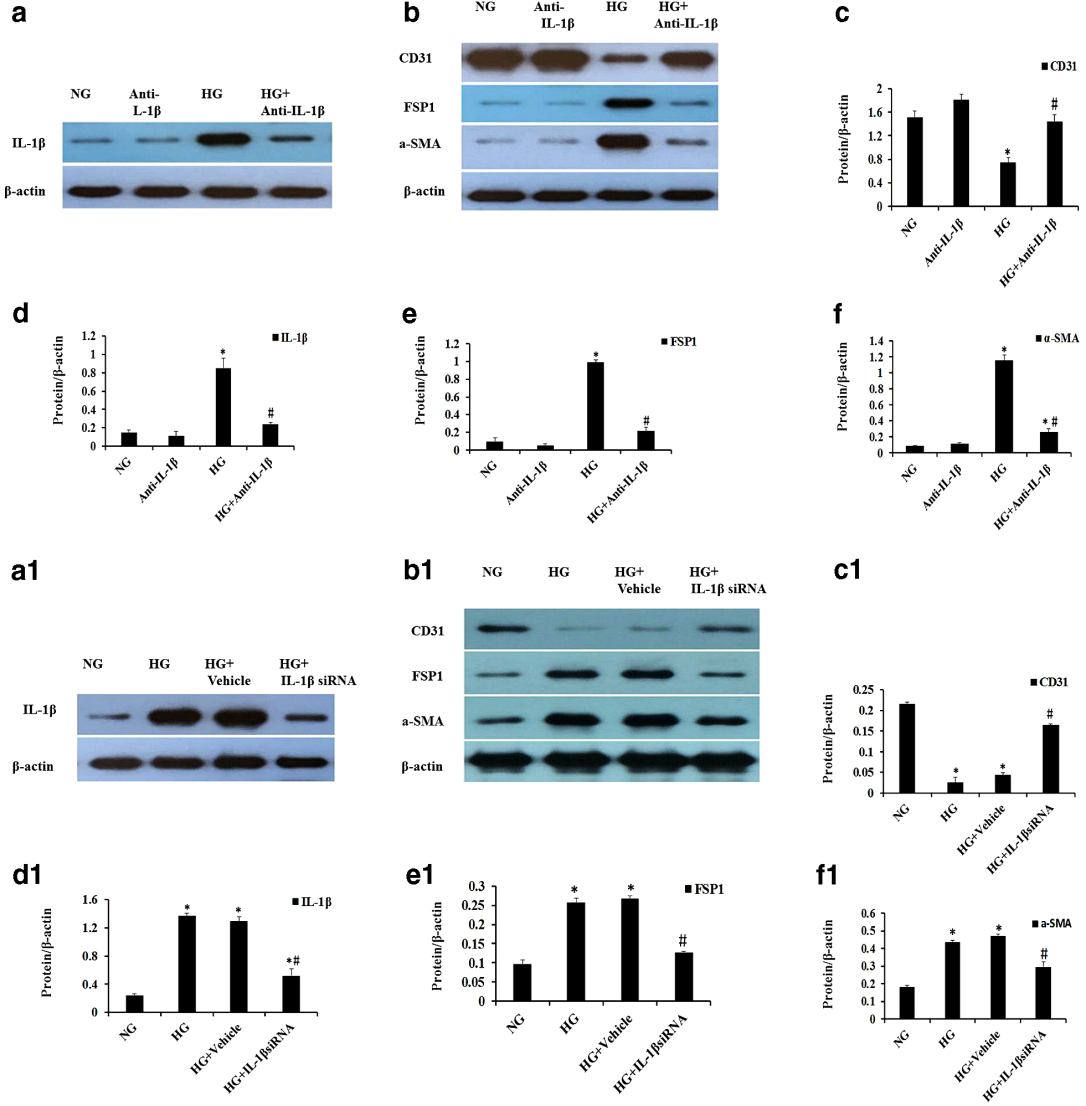
The influence of blocking IL-1β treatment on the protein expressions of CD31, FSP1, a-SMA, and IL-1β. (**a**–**f**) HAECs were incubated for 48 h with anti-IL-1β antibodies (1000 ng/ml) in the presence of NG or HG. (**a1**–**f1**) We performed gene-silencing experiments using transfection with siRNA specific for IL-1β. The protein expressions of IL-1β, CD31, FSP1 and α-SMA were assessed by western blotting. The data are expressed as the mean ± SD. Experiments were repeated at least three times. *NG* normal glucose (5.5 mM), *HG* high glucose (30 mM). Anti-IL-1β: anti-IL-1β antibodies (1000 ng/ml). *P < 0.05 vs. NG or anti-IL-1β, ^#^ *P* < 0.05 vs. HG or HG +Vehicle

We further evaluated the influence of IL-1β on the markers related with the phenotypic transition in HAECs. As a classic antagonist, anti-IL-1β antibodies were used to block IL-1β pathway in vitro. As shown in Fig. 6 a–f, the treatment of anti-IL-1β antibodies largely prevented FSP1, α-SMA and IL-1β expression in the protein levels and improved CD31 expression. Consistently, the treatment of IL-1β siRNA again suggested that the inhibition of IL-1β prevented HG-triggered mesenchymal transition of HAECs (Fig. 6a1–f1).

### Neutralizing IL-1β improved pathological changes in HAECs

**Fig. 7:**
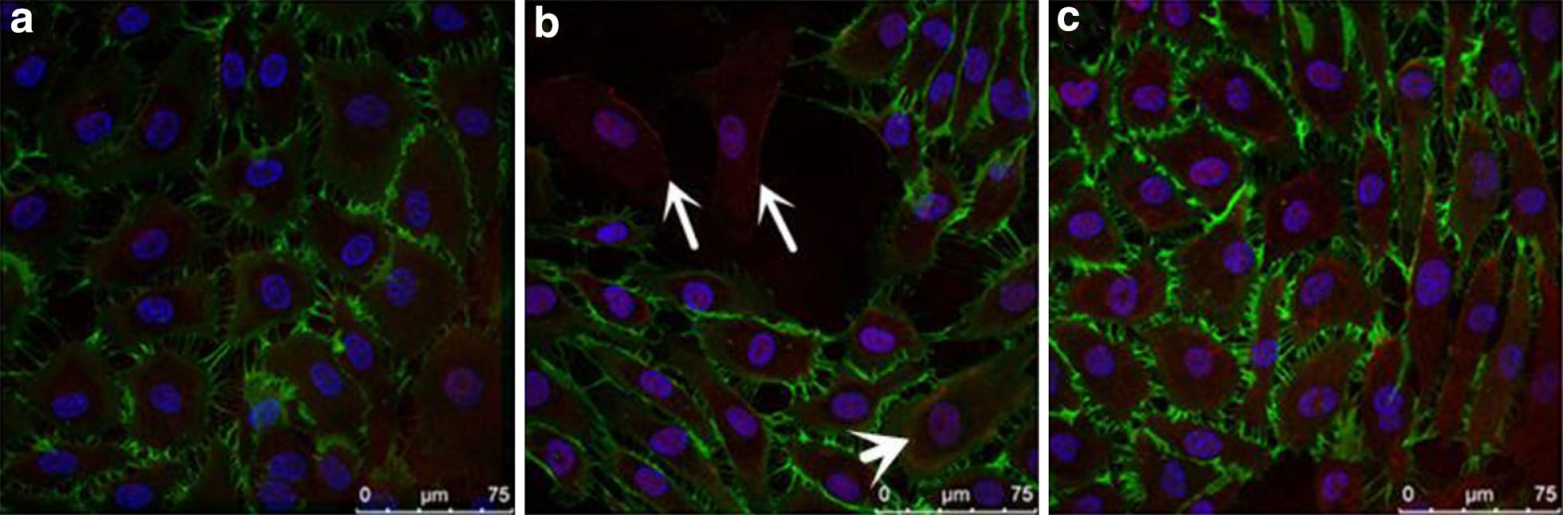
Anti-IL-1β antibodies treatment inhibited high glucose-induced phenotypic transition of HAECs, asassessed by laser scanning confocal microscopy. Representative immunofluorescence images showing CD31 (*green*), FSP1 (*red*) labeling and DAPI (*blue*) stains nuclei. **a** Normal ECs monolayers displayed a cobble stone morphology. **b** A merge of the three images revealed some cells populations that acquired a *spindle-shaped* morphology and lost CD31 expression (*white arrow heads*). **c** The administration of anti-IL-1β antibodies treatment caused a reduction of these changes (*P* < 0.05). **a** normal glucose (5.5 mM) group, **b** high glucose (30 mM) group for 48 h; **c** treatment with a high glucose concentration (30 mM) + anti-IL-1β antibodies (1000 ng/ml) treatment for 48 h. Experiments were repeated three times. *Scale bar*, 75 μm.*P < 0.05 vs.HG

Based on confocal microscopic analysis, normal ECs monolayers displayed a cobble stone morphology (Fig. 7a). In our study, the HAECs, which were exposed to HG for 48 h, exhibited profound changes with cells becoming elongated and spindle-shaped and lost cobblestone morphology. And we performed labelling experiments using anti-CD31 (green) and anti-FSP1 (red, also termed S100A4) antibodies. An analysis of FSP1/CD31 double labelling revealed that some cells acquired FSP1 staining and lost CD31 staining (Fig. 7b, white arrow heads), which suggested that phenotypic transition occurred in HAECs.

**Fig. 8:**
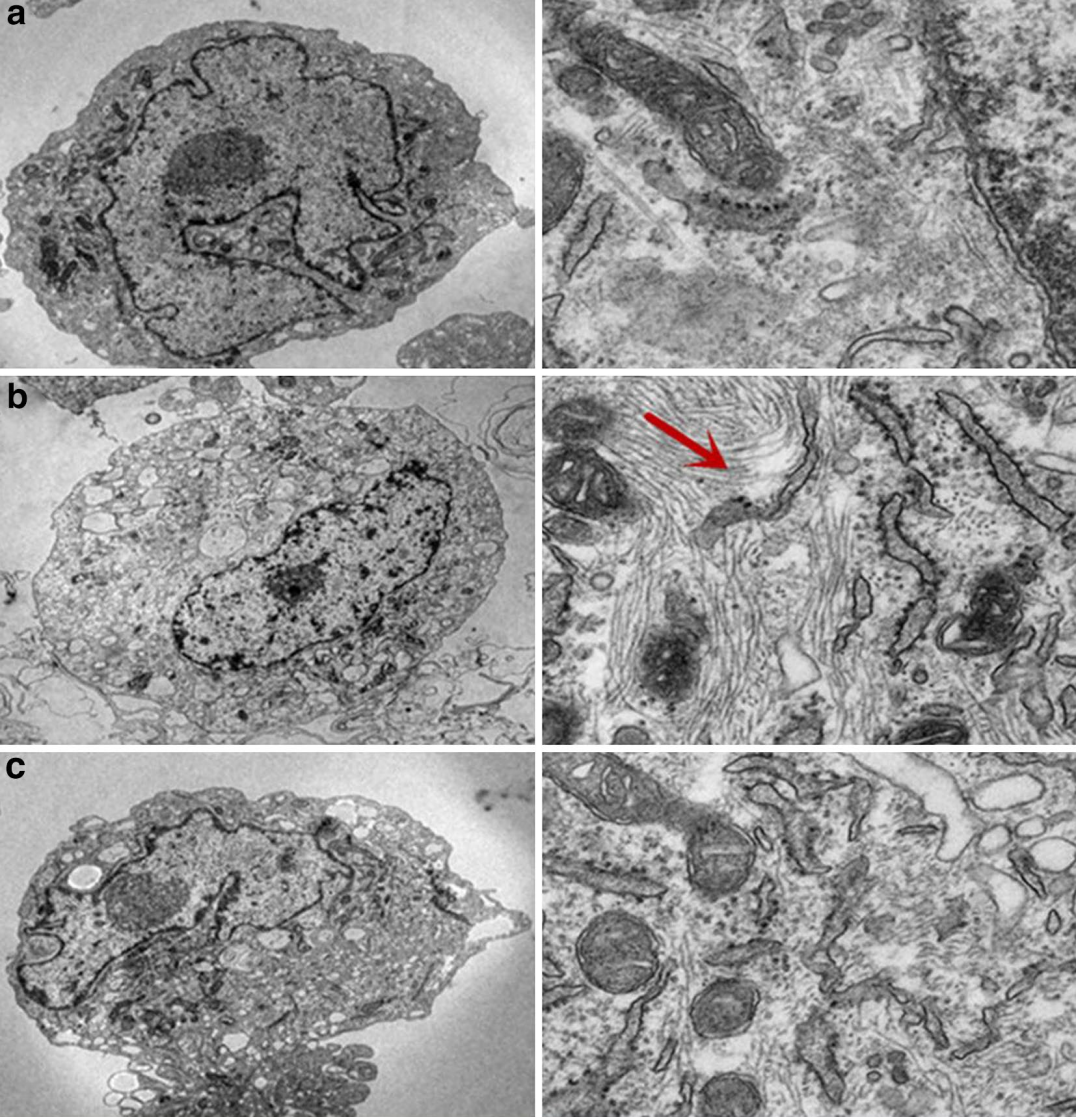
Anti-IL-1β antibodies inhibited high glucose-induced phenotypic transition of HAECs, as assessed by transmission electron microscopy. Transmission electron microscopy depicts the change in cellular ultrastructure following HG (30 mM) exposure (left magnification ×10,000,vs right magnification ×40,000 in the same group). **a** It can be seen that normal HAECs present with few microfilaments and a rough endoplasmic reticulum. **b** After exposure to HG, microfilamentation and a swollen rough endoplasmic reticulum appeared in the cytoplasm. **c** These changes were attenuated by treatment with anti-IL-1β antibodies. **a** normal glucose (5.5 mM) group, **b** high glucose (30 mM) group for 48 h; **c** treatment with a high glucose concentration (30 mM) + anti-IL-1β antibodies (1000 ng/ml) treatment for 48 h

Parallel with the findings above, electron microscopy analysis of the NG group demonstrated that the EC therein exhibited normal structures (Fig. 8a). In contrast, the HG group that was treated with HG (30 mM) for 48 h exhibited endothelial protrusions, a significantly roughened endoplasmic reticulum, and microfilamentation (Fig. 8b, red arrow head). Interestingly, these changes were attenuated by the treatment with anti-IL1β antibodies (Fig. 7c *P* < 0.05 with Fig. 8c).

## Discussion

A hallmark of diabetic vascular pathology is EC damage [26]. EC damage is considered to be the early site, accelerates atherosclerosis and subsequently causes cardiovascular events [27]. The mechanisms behind this phenomenon are probably multifactorial including the polyol pathway, activation of PKC, increased oxidative stress, advanced glycation end (AGE) product formation, and inflammation [25, 28, 29]. Meanwhile, these influencing factors are related to each other, rather than isolated. For instance, the oxidative stress and AGE can result in inflammation [30]. Moreover, recent studies revealed that PKCβ activation could promote EC inflammation and cause EC damage in diabetes [28, 31, 32]. The inflammation, including cytokines, also plays an important role in EC damage in diabetes.

A large body of evidence emphasized that the low-grade chronic inflammatory activation, as a potential contributor to EC damage, increased the vascular diseases [33]. Indeed, previous studies suggested that the diabetes was an inflammatory disease, which was mainly based on the increased plasma concentrations of IL-6, IL-1, and TNF-α [3, 11, 34]. And the increased cytokines, serving as early markers for vascular inflammation, could be responsible for EC damage in diabetes [15]. Especially, IL-1β, one of the earliest activated cytokines in the injury tissues, was reported to induce EC damage in isolated rat mesenteric micro-vessels [35, 36]. Recent study suggested that EC damage in diabetes might be linked to the mechanisms triggered by IL-1β and recovered by IL-1 receptor antagonist [15]. Moreover, the enhanced expression of IL-1β in HG conditions was described in human monocytes and macrophages [37, 38], pancreatic islets [39], and HAECs [40], meanwhile, the up-regulation of IL-1β was also described in the retina and retinal vessels from diabetic rats [10]. In the present study, we demonstrated that HG induced IL-1β expression, which was consistent with the observation by Asakawa et al. [40]. Our findings indicated that IL-1β induced its own synthesis at dose-dependent manner (data not shown), as was known to be the trigger and amplifier of inflammation [10]. These results implied that EC was also a major source of IL-1β under HG conditions. Simultaneously, we observed elevated PKCβ level accompanied by increased IL-1β. Furthermore, the next findings showed that IL-1β production was up-regulated by PMA and down-regulated by PKCβ inhibitor in HAECs exposed to HG. Therefore, the results indicated that PKCβ activation may mediate IL-1β production in HAECs with HG exposure. Thus, it seemed plausible that IL-β and PKCβ were involved in HG-induced EC damage. However, the mechanism underlying the effect of IL-β on EC damage in diabetic is incompletely understood.

Increasing evidence showed that when exogenous IL-1β stimulated retinal, human intestinal and dermal micro-vascular EC,respectively, EC underwent EndMT [16–19, 41]. EndMT was first described in the embryonic heart [42], involved in murine transplant arteriopathy, vascular cerebral cavernous malformations and vascular graft remodelling [23, 43, 44]. Our previous work also demonstrated that angiotensin II was partially involved in the process of EndMT, contributing to HG-induced EC damage [24, 25]. The process could be regulated by multiple factors such as angiotensin II and endothelin-1 under HG conditions. Whether HG-mediated local inflammation, including IL-1β, is involved in the mesenchymal phenotype of EC has not been completely elucidated. Our results demonstrated that HAECs, incubated with HG or IL-1β, developed a series of phonotypical changes and the over expressions of FSP1 and α-SMA, which suggested the occurrence of a phenotypic transition in HAECs. Additionally, HG and IL-1β synergistically resulted in profound similar changes. Moreover, changes above were attenuated by the treatment of anti-IL-1β antibodies or IL-1β siRNA. Together with the results of increased IL-1β, we believed that IL-1β may mediate the phenotypic transition in HAECs with HG treatment.

However, the inflammation system is far more complex than the IL-1 system and it is expected that other anti-inflammatory treatments such as anti-TNF, or in combination, will have additional and complementary effects on diabetic vasculopathy.

## Conclusions

In summary, our study demonstrated that HG induced phenotypic transition in HAECs, which was mediated in part by the IL-1β production. The blockade of IL-1β production may prevent the phenotypic transition in HAECs exposured to HG. In addition, PKCβ activation may be responsible for upregulation of IL-1β by HG. Our findings implied that IL-1β or PKCβ pathway blockade would be a novel and effective strategy to control diabetic vasculopathy.

## Abbreviations

EC: endothelial cell
IL-1β: interleukin-1β
NG: normal glucose
HG: high glucose
MN: mannitol
PKCβ: protein kinase C-β
HAECs: human aortic endothelial cells
PMA: phorbol12-myristate 13-acetate
LY: LY317615
FSP1: fibroblast-specificprotein1
α-SMA: α-smooth muscle actin
siRNA: small interferingRNA
EndMT: endothelial-to-mesenchymal transition
ELISA: enzyme linked immuno-sorbent assay
TEM: transmission electron microscopy
